# Recent insights into the role of innate immunity in lupus

**DOI:** 10.1093/hmg/ddaf066

**Published:** 2025-06-05

**Authors:** Lauren A Robinson, Virginia Pascual

**Affiliations:** Drukier Institue for Children’s Health, Weill Cornell Medical College, 513 East 69th Street, New York, NY 10021, United States; Department of Pediatrics, Weill Cornell Medical College, 525 East 68th Street, New York, NY 10021, United States; Hospital for Special Surgery, 535 East 70th Street, New York, NY 10021, United States; Drukier Institue for Children’s Health, Weill Cornell Medical College, 513 East 69th Street, New York, NY 10021, United States; Department of Pediatrics, Weill Cornell Medical College, 525 East 68th Street, New York, NY 10021, United States

**Keywords:** Lupus, Toll like Receptors, Inflammasome, Myeloid cells, Microbiome

## Abstract

Systemic Lupus Erythematosus (SLE) is a complex autoimmune disorder characterized by loss of self-tolerance to nucleic acids, resulting in multisystem inflammation and organ damage. The genetic underpinning of SLE spans from common risk variants with modest effect sizes to rare monogenic mutations with high penetrance. Recent advances in next-generation sequencing and transcriptomic profiling have illuminated the central role of innate immune pathways in disease pathogenesis. This review synthesizes emerging evidence regarding innate immunity in SLE, with emphasis on toll-like receptor (TLR) signaling and regulatory mechanisms, NLRP3 inflammasome activation, myeloid cell dysregulation, and microbiome-immune interactions. Understanding these pathways provides a foundation for developing targeted therapeutics that may offer precision medicine approaches for this heterogeneous disease.

## Graphical Abstract

**Graphical Abstract:**
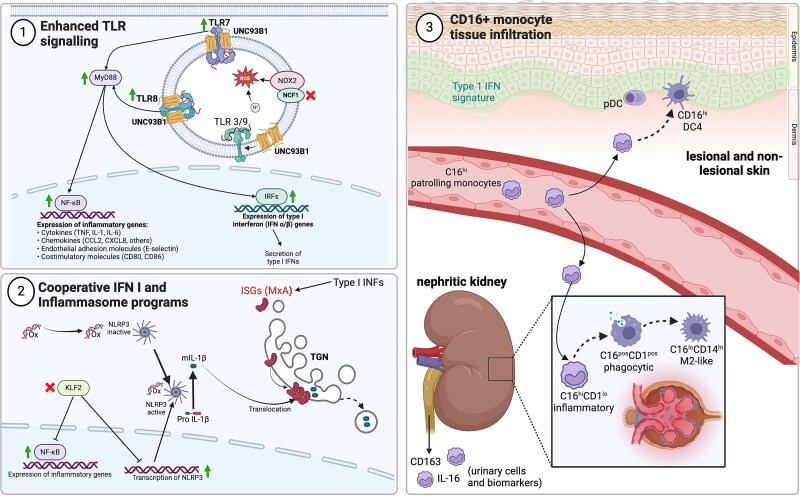

## Introduction

Systemic Lupus Erythematosus (SLE), a prototypic autoimmune disease, primarily affects women of child-bearing potential, but up to 20% of patients are diagnosed in childhood. The prevalence of SLE generally ranges from 20–150 per 100 000 worldwide, disproportionately impacting Black and Hispanic women [1–3]. Clinical disease manifestations vary widely from the milder photosensitive rashes and articular manifestations to the more severe internal organ manifestations including nephritis and neuropsychiatric disease. Pathogenic hallmarks of disease include production of autoantibodies to nuclear antigens, activation and consumption of complement, and interferon signaling. It is generally believed that environmental triggers such as ultraviolet radiation and viral infections lead to breaks in immune tolerance in genetically susceptible hosts to cause disease. The mainstays of treatment include glucocorticoids, antimalarials, and broadly-acting immunosuppressants, often resulting in significant toxicity and inadequate disease control. The first targeted biologic medication approved for treatment of SLE, a monoclonal antibody to the B-cell activator factor BAFF (Belimumab), became available in 2011 [4, 5]. Although this targeted B-cell directed therapy has shown efficacy in large trials and is a useful adjunct in SLE treatment, it is ineffective for many patients, highlighting the heterogeneity of disease pathogenesis.

**Table 1:** Summary of genes relevant to innate immunity that are associated with development of SLE phenotypes, grouped by innate immune pathway.

Innate Immune Pathway	Common Variants	Rare Variants	Phenotypic Features and Overlapping Diagnoses Associated with Rare Variants
Clearance of apoptotic debris and immune complexes	ATG5, DRAM1 FcGR2A, FcGR3A, ITGAM [11–14]	C1Q, CIQR/S, C2, C4A/B [15]	Neuroinflammation, Immunodeficiency, Nephritis, photosensitivity
ROS production (phagocytic function, alkalinization of endolysosomes)	NCF1, NCF2 [16–18]	CYBB [15, 19, 20]	Chronic Granulomatous Disease, Autoimmunity
Nucleic acid degradation and editing		DNASE1, DNASE1L3 [21, 22] RNASEH2, RNASEH2B, RNASEH2C TREX1, SAMHD1, ADAR1 [15, 23–26]	Anti-nucleosome antibodies, Sjogrens Syndrome Neuroinflammation, Chilblain Lupus, Aicardi Goutières Syndrome (AGS)
Nucleic acid sensing/TLR signaling	IFIH1 (MDA5) IRF3, IRF5, IRF7, IRF8, IRAK4 [11–14, 18] TLR7 [27, 28]	IFIH1(MDA5) [29] TLR7, UNC93B1 [30–38]	Neuroinflammation, AGS, IgA deficiency Neuroinflammation, Chilblain Lupus, AGS, Autoimmune Cytopenia
Type 1 interferon Signaling	STAT4, TYK2 SPP1 (OPN) [18, 39, 40]	ACP5 (TRAP) [41, 42]	Spondyloenchondrodysplasia (SPENCD), Immune Dysregulation
NF-kB and inflammasome signaling	TNIP1, TNFAIP3, HIC2/UBE2L3, SLC15A4, KLF2 [40, 43, 44]	TNFAIP3 [38]	Haploinsufficiency of A20 (HA20)
Polyamine Metabolism (Neutrophil homeostasis and function)		SAT-1 [45]	X-linked disease in males, Nephritis

Beyond the well-documented type I interferon (IFN) signature in peripheral blood and affected tissues, inflammatory cascades involving neutrophils and monocytes play significant roles [6]. The pathogenic relevance of type I IFN signaling has been validated in the phase III trials of anifrolumab, an IFN receptor antagonist approved by the FDA in 2021. Notably, approximately half of enrolled patients failed to demonstrate improvement beyond standard care in spite of displaying baseline IFN signatures [7–10]. These data emphasize the complexity of IFN-related immunopathology and the need for deeper mechanistic understanding to develop more effective therapeutic strategies.

Advances in genomic technologies have accelerated the identification of both rare monogenic and common polygenic variants associated with SLE. Beyond B-cell intrinsic defects, numerous SLE-associated genes participate in innate immune pathways, including complement cascade components, reactive oxygen species (ROS) production, nucleic acid metabolism and sensing, and interferon signaling pathways (Table 1). Simultaneously, transcriptomic profiling at bulk and single-cell resolution in blood and target tissues is unveiling novel dysregulated immune networks in patients without identifiable monogenic etiologies.

In this review, we summarize recent genetic and immunophenotypic evidence linking nucleic acid sensing and myeloid cell pathways to SLE pathogenesis, with additional consideration of emerging data on microbiome-immune interactions in lupus.

## Paying the toll for nucleic acid sensing in SLE

Toll like receptors (TLRs) are transmembrane receptors that play a key role in innate immunity through the sensing of pathogen and damage associated molecular patterns. TLRs 3, 7, 8 and 9 reside in the endoplasmic reticulum and translocate into the membranes of endosomes and lysosomes, where they recognize NAs from endocytosed viruses or surrounding tissue damage. Signaling through endosomal TLRs plays a critical role in anti-viral and anti-microbial responses via upregulation of inflammatory cytokines and type I IFNs. TLR7, which recognizes single stranded RNA, has been implicated in SLE pathogenesis broadly as a sensor of endogenous NAs upstream of type I IFN production, and as an inducer of intrinsic B cell activation and autoreactivity. [46–48] The roles of TLR8 and TLR9 in SLE pathogenesis are less clear. In murine lupus models, TLR9 is necessary for the formation of anti-dsDNA antibodies, but its knockout may worsen disease [49–51]. Thus, TLR9 confers protection in various lupus models as a result of its capacity to restrain TLR7 responses in a cell-type specific fashion. Additional studies of these complex TLR interactions in humans are needed, however, to determine their potential role in disease [52].

The contribution of the RNA sensor TLR8 to autoimmunity has proven particularly challenging, due to interspecies differences in expression patterns and ligand specificity between humans and mice [53]. While human TLR8 LOF mutations remain unreported, GOF mutations have been identified in six unrelated male patients—five exhibiting somatic mosaicism. Importantly, these individuals manifested not with SLE or type I interferonopathy, but rather with a distinct phenotype characterized by infection susceptibility, compromised humoral immunity, and neutropenia [54]. This clinical presentation stands in contrast to predictions derived from transgenic mouse models overexpressing human TLR8, which develop increased susceptibility to collagen-induced arthritis [55]. Despite overlapping signal transduction pathways between TLR7 and TLR8, the absence of TLR8 expression in human B cells may account for the unexpected phenotypic divergence observed between GOF mutations affecting these phylogenetically related pattern recognition receptors [53].

### TLR7 GOF: An unequivocal link to SLE

TLR7 duplication is a well-known cause of murine lupus, and various TLR7 risk alleles have been identified over the last two decades in patients with SLE [27, 28, 30]. Recent studies conclusively demonstrated that GOF TLR7 variants directly cause autoimmunity in humans [31, 36, 37]. The de novo Y264H variant, identified in a female with childhood-onset SLE, enhances TLR7 affinity for guanosine and amplifies downstream signaling in response to endogenous ligands—an effect sufficient to recapitulate disease when introduced into murine models [31]. Subsequently identified GOF variants (F507L, F507S, and P267L) have been observed in pediatric SLE cohorts [31, 36, 37]. While the precise molecular mechanisms underlying enhanced TLR7 signaling remain under investigation, these mutations cluster at the TLR7 homodimer interface, potentially facilitating ligand-independent receptor dimerization and constitutive activation.

TLR7 GOF mutations appear to contribute to human SLE pathogenesis through both cell intrinsic and extrinsic pathways. In mice carrying the human Y264H mutation, increased B-cell intrinsic signaling leads to increased germinal center formation as well as to extrafollicular expansion of autoreactive B cells, which are relevant to both murine and human SLE pathogenesis [31, 48]. Concurrently, increased production of type I IFN by plasmacytoid DCs (pDCs) expressing constitutively active TLR7 induce both innate and adaptive immune dysregulation, including heightened antigen presentation by conventional DCs (cDCs), polarization towards Th1 responses, and differentiation of mature B cells into antibody secreting plasma cells [56–58]. The contribution of TLR7 GOF to disease through the activation of tissue resident and/or infiltrating myeloid cells remains however to be further investigated.

Interestingly, several patients with TLR7 GOF presented with refractory immune-mediated thrombocytopenia (ITP) and anemia [31, 59]. Beyond lymphoid and myeloid cells, TLR7 is expressed in human platelets, and specialized bone marrow monocyte-derived ‘hemophagocytes’ have been shown in mice to engulf erythrocytes and platelets in a TLR7/TLR9-dependent manner [60]. Platelet-intrinsic TLR7 activation also promotes formation of platelet–neutrophil complexes and enhances neutrophil extracellular trap formation (NETosis) [61], a pathway linked to SLE pathogenesis [62–64].

Additionally, TLR7 expression in human microglia may explain the severe neurologic manifestations, including chorea, cerebral vasculitis and NMDA receptor encephalitis, found in SLE patients carrying TLR7 GOF mutations. These variants, however, have also been identified in patients with neuroinflammatory phenotypes, such as Neuromyelitis Optica or Aicardi Goutières, in the absence of systemic autoimmunity [36]. Thus, while TLR7 GOF is sufficient to cause SLE phenotypes in both humans and experimental models, the phenotypic heterogeneity likely reflects unique impacts of specific mutations on the diverse hematopoietic and tissue-resident cell populations expressing this pattern recognition receptor.

### TLRs, X chromosome inactivation and SLE

Among human TLRs, TLR7 and TLR8, are encoded on the X chromosome. To balance gene expression in males and females, the long non-coding RNA (lncRNA) XIST stochastically induces X chromosome inactivation (XCI) during female embryonic development. XIST activity, however, is required during post-embryonic life in human B cells, where it maintains silencing of a subset of X-linked immune genes, including TLR7. Escaping from XCI could potentially result in bi-allelic gene expression of these genes. In fact, single-cell transcriptomic data from female SLE patients revealed XIST dysregulation affecting CD11c^+^ atypical or double negative 2 memory B cells (ABCs or DN2 cells, respectively), which are known to expand in SLE and differentiate into antibody-secreting cells upon TLR7 ligation [65]. While this observation could explain female-biased SLE susceptibility, definitive evidence that XCI escape leading to increased TLR7 copy number in B cells drives disease pathogenesis remains to be established. Furthermore, the potential contribution of dysregulated XCI affecting both TLR7 and TLR8 expression within the myeloid compartment, a pathway demonstrated to promote lupus-like disease in murine models with altered XIST function [66], would require further investigation in human SLE.

### TLR modulators and SLE

Following the discovery that TLR7 GOF variants are linked to human SLE, additional molecules involved in TLR trafficking and degradation, most notably UNC93B1 and NCF1, have been implicated in SLE. We will next discuss emerging data on the relevance of these pathways in SLE pathogenesis.

#### UNC93B1

Endosomal TLRs originate in the ER prior to trafficking through the Golgi and ultimately to endosomes with the help of molecular chaperones like UNC93B1 [67–70]. Within endosomes, TLRs are cleaved to their active form, allowing for ligand binding [71–73]. UNC93B1 serves multifaceted roles beyond trafficking, participating in endosomal stabilization and signal termination through diverse molecular mechanisms [34, 74, 75]. Further complexity arises from differential binding affinities between UNC93B1 and various endosomal TLRs, with TLR9 demonstrating preferential interaction. Notably, mutations disrupting this preferential binding lead to TLR7-mediated systemic autoimmunity in murine models [76].

In humans, UNC93B1 deficiency mimics TLR3 deficiency, conferring susceptibility to herpes simplex virus-1 (HSV-1) encephalitis through impaired production of type I and type III IFNs [77]. However, large scale mutational analysis in mice and characterization of human GOF UNC93B1 variants support that TLR7 and TLR8 are the main targets of UNC93B1-mediated regulation [32–35, 78]. This suggests a model where TLR3 and TLR9 may require dissociation from UNC93B1 within endosomal compartments to facilitate ligand binding and signal transduction, while activated TLR7 and TLR8 isoforms maintain stable complexes with this chaperone[75]. Collectively, the differential effects of UNC93B1 variants on specific TLR signaling pathways underscore the intricate molecular interactions governing this regulatory system and might explain the diverse clinical manifestations associated with UNC93B1 mutations, ranging from SLE to chilblain lupus and neuroinflammatory disorders.

Not only do the nuanced interactions between UNC93B1 variants and endosomal TLRs influence the resulting phenotypes, but they also allow for a spectrum of disease inheritance patterns. These include autosomal dominance with complete penetrance, autosomal dominance with incomplete penetrance, and an additive semi-dominance in which heterozygous carriers manifest only subclinical immune dysregulation [33, 34]. Additionally, a specific UNC93B1 allele (V117L) predominantly found in the South coast Han population of East Asia confers a remarkable 17.9-fold increased risk for childhood-onset SLE without displaying classical Mendelian inheritance patterns [32].

#### NCF1

TLR7 signaling is regulated by endosomal pH, requiring an acidic environment for proteolytic cleavage to its active form [71, 72, 79]. Neutrophil Cytosolic Factors 1 and 2 (NCF1and NCF2) are cytosolic subunits of the phagocyte NADPH oxidase complex (NOX2) that elevate phagosomal pH, thereby modulating endosomal processing [80, 81]. Patients lacking functional NCF1 develop chronic granulomatous disease (CGD), an inborn error of immunity frequently complicated by autoinflammation and autoimmunity, including SLE [19]. Polymorphisms in NCF1 and NCF2 genes resulting in decreased but not absent ROS production have been identified as SLE risk alleles in various populations, while increased NCF1 copy number confer protection against SLE [16, 17, 39, 82].

While NCF1 and NCF2 variants causing reduced ROS generation were initially proposed to contribute to SLE pathogenesis through enhanced neutrophil extracellular trap (NET) formation and impaired macrophage efferocytosis [83–85], recent evidence reveals a critical role for NCF1 in regulating TLR7 signaling within both pDCs and B cells [86–88]. NCF1 likely extends this regulatory function to monocytes, as cell-specific ablation of NADPH oxidase in either monocytes or B cells within TLR7-dependent murine lupus models results in amplified TLR7 signaling and exacerbated disease phenotypes [87].

In-vitro studies show that pDCs harboring mutations that impair NCF1 endosomal localization and phospholipid binding exhibit increased endosomal acidification, TLR cleavage and response to TLR ligands [86]. NCF1 also plays an intrinsic role in B cells, as NOX2-deficient primary B cells show enhanced signaling upon endosomal TLR ligation [87]. These findings support similar mechanisms linking variants in NCF1 and UNC93B1 with enhanced TLR7 signaling in various immune cells, ultimately promoting autoimmunity.

## Myeloid cells: Instigators or responders?

Myeloid cells play key roles in SLE pathogenesis, among others via production of type I IFN, defective clearance of apoptotic debris, NETosis and release of oxidized nucleic acids [62, 89–91]. These these innate cells have traditionally been thought of as amplifyers of the inflammatory processes already set in motion rather than initiators of disease. However, this paradigm is evolving, as longitudinal immunophenotyping studies in large patient cohorts have identified distinct SLE subgroups wherein disease activity—particularly nephritis—correlates with elevated myeloid and neutrophil transcriptional signatures, suggesting heterogeneous contributions of these cell populations across different patient subsets and disease phases [92]. In this section, we highlight the discovery of the first monogenic SLE variant that positions neutrophils as primary instigators of disease pathogenesis, along with recent advances characterizing myeloid cell phenotypes and functions in both peripheral blood and affected tissues of SLE patients.

### SAT-1 LOF

Spermidine/spermine N1-acetyltransferase (SAT-1), encoded by the X-linked SAT1 gene, functions as the rate-limiting enzyme in polyamine catabolism. Polyamines—ubiquitous natural organic compounds—control cellular homeostasis, proliferation, differentiation, and programmed cell death [93]. A breakthrough discovery identified LOF mutations in SAT1 in two unrelated kindreds exhibiting X-linked inheritance of SLE. Subsequent generation of murine knock-in models recapitulated the lupus phenotype, establishing disrupted polyamine metabolism as sufficient to induce autoimmunity [45]. Although the exact mechanisms are not fully understood, SAT1-deficient neutrophils exhibit multiple functional abnormalities, including impaired autophagy, spontaneous NET formation with release of oxidized mitochondrial DNA, and defective clearance of apoptotic cells. Beyond these innate immune perturbations, the murine model demonstrated reduced regulatory T cell (Treg) frequency, potentially contributing to immune dysregulation. Complementary clinical studies have documented decreased levels of specific polyamines in SLE patients, further supporting a role for aberrant polyamine metabolism in disease pathogenesis through dual mechanisms affecting both neutrophil function and Treg homeostasis [45, 94].

### The NLRP3 Inflammasome and type 1 IFN: Unlikely partners in SLE pathogenesis?

The role of IL-1β and the NLRP3 inflammasome in SLE pathogenesis remains controversial, with murine models yielding inconsistent results [95–98]. In vitro studies demonstrated that immune complexes (ICs), NET components, and complement activation products can trigger NLRP3 inflammasome activation in monocytes, contributing to organ damage in vivo [99–101]. Consistent with these findings, monocytes from lupus patients exhibit simultaneous activation signatures of both inflammasome and type I interferon pathways, representing a breakdown of the well-established counter-regulatory relationship between these inflammatory cascades [102–105]. However, while transcriptional evidence links inflammasome activation to SLE, genetic associations remain limited. A single risk allele in KLF2—a transcription factor modulating NF-κB signaling—recently established a genetic connection between inflammasome regulation and SLE susceptibility in Asian populations [43, 44, 106]. Here, we highlight recent advances in understanding the potential contributions of NLRP3 inflammasome and IL-1β to human lupus pathogenesis.

Transcriptomic analyses across ethnically diverse SLE cohorts have consistently identified upregulation of myeloid inflammatory pathway transcripts, particularly in patients with lupus nephritis (LN) [92, 107]. Mechanistically, prolonged priming of SLE monocytes with type I interferons or patient sera enhances inflammasome activation, partly through IRF1-dependent CASP1 induction [102]. Single-cell RNA sequencing of peripheral blood monocytes from pediatric SLE patients revealed two expanded IFN-stimulated gene (ISG) high monocyte subclusters in individuals with high disease activity, one of which co-expressed ISGs and IL1B transcripts [108].

More recent studies linked this cellular phenotype to the capacity of monocytes to opsonize mitochondria-retaining mature red blood cells (Mito+ RBCs), a characteristic feature of SLE [109]. In this context, release of erythrocyte-derived mitochondrial DNA (mtDNA) and RNA (mtRNA) into the monocyte cytosol triggers the release of short monocyte mtDNA fragments that bind and activate the NLRP3 inflammasome. Subsequent IL-1β release occurs through a gasdermin D (GSDMD)-independent pathway but requires cooperative signaling with an interferon-stimulated gene product. Specifically, oligomerization of the IFN-induced protein MxA on the trans-Golgi network (TGN) membrane facilitates translocation of mature IL-1β (mIL-1β) into this compartment. Crucially, mIL-1β secretion proceeds without pyroptosis or any other form of monocyte death [110].

These findings suggest that highly inflammatory monocyte populations exhibiting uniquely synergistic IFN and inflammasome activation programs may contribute to disease pathogenesis in a subset of SLE patients, potentially identifying candidates who might benefit from IL-1β-targeted therapeutic interventions.

### Going where the action is: Immunophenotyping myeloid cells in tissues

Studies in murine lupus models have revealed remarkable heterogeneity among myeloid populations within the nephritic kidney. In human lupus nephritis (LN), both tissue-resident and infiltrating myeloid cells represent a substantial component of affected kidney tissue, and myeloid-derived proteins rank among the most sensitive urinary biomarkers for LN activity and progression [111–113].

Despite these advances, comprehensive characterization of myeloid cell diversity and functional significance in human LN remains incomplete. A fundamental unresolved question concerns whether these myeloid populations are disease initiators or amplifiers of pre-established inflammatory cascades in SLE pathogenesis. This distinction carries profound implications for therapeutic targeting strategies. In this section, we synthesize recent breakthroughs in characterizing myeloid populations within human SLE-affected tissues and critically evaluate emerging evidence regarding their role in disease initiation versus propagation.

#### The myeloid compartment in the lupus kidney

In murine lupus models, blood monocytes infiltrate the renal tissue following the appearance of lymphoid aggregates and immune complex deposition. These infiltrating monocytes exhibit dysregulated activation profiles and participate in multiple processes including phagocytosis, inflammatory amplification, and tissue repair/remodeling [114–116]. Recent single-cell transcriptomic analyses of kidney tissue from patients with LN provided unprecedented resolution of the myeloid compartment diversity in human disease [117]. These studies identified five distinct myeloid cell clusters: conventional dendritic cells (DCs); kidney-resident macrophages (characterized by upregulated interferon-stimulated genes, anti-inflammatory mediators, and TLR signaling inhibitors compared to healthy controls); and three probable infiltrating monocyte-derived populations. Trajectory analyses suggest in situ phenotypic evolution across these infiltrating populations, progressing from an inflammatory CD16+ patrolling monocyte phenotype through a transitional phagocytic monocyte state, and ultimately differentiating into alternatively activated M2-like scavenging monocytes/macrophages [117]. Complementary spatial transcriptomic studies of renal biopsies from pediatric patients with proliferative LN recently confirmed significant glomerular myeloid infiltration, as observed in murine models [118]. However, the relative contributions of these myeloid populations to inflammatory tissue damage versus resolution and repair in human disease remain incompletely defined.

A critical unresolved question is whether these infiltrating monocytes actively initiate glomerular inflammation or primarily respond to and amplify pre-existing inflammatory processes triggered by immune complex deposition and lymphocytic infiltration. Notably, the infiltrating monocyte subclusters identified in kidney tissue—particularly inflammatory CD16+ monocytes—have also been detected through single-cell transcriptomic analysis of LN urine sediment [117]. Furthermore, urinary proteomics identified IL-16 and CD163 as biomarkers that correlate most strongly with nephritis activity index and treatment response [119, 120]. CD163, predominantly expressed by infiltrating myeloid cells in LN kidneys, represents a particularly promising biomarker [120]. These findings suggest that urine might provide useful information for diagnosis and follow up of LN to complement and hopefully in the future replace the need for invasive procedures.

#### Myeloid cells in the lupus skin

In situ studies of skin lesions from patients with systemic and cutaneous lupus erythematosus (CLE) provide important insights into the immunological alterations that may precede and contribute to tissue inflammation. Single cell transcriptomic analysis of both lesional and non-lesional lupus skin confirmed elevated type I IFN signatures, particularly in basal keratinocytes and fibroblasts, as well as pDC infiltration at the dermo-epidermal junction [121–123]. A striking finding is the expansion of CD16+ DCs, a recently characterized DC subset (DC4) that shares considerable transcriptional overlap with non-classical monocytes and exhibits enrichment for ISG expression [124, 125]. Trajectory analyses combining cells from both peripheral blood and skin support the hypothesis that blood non-classical CD16+ monocytes are precursors of CD16+ DCs found in the skin. Spatial transcriptomic analysis localized the majority of these DCs to the superficial dermis near the dermo-epidermal junction, where they are poised to interact with endothelial cells and ISG^hi^ fibroblasts. It is therefore possible that blood CD16+ monocytes infiltrate the skin, where they differentiate into CD16+ DCs and acquire a pro-inflammatory phenotype following exposure to the keratinocyte-derived type I IFN family member IFNκ [124].

Taken together, single cell transcriptomic analyses of lupus tissues reveal a consistent pattern of non-classical monocyte infiltration and subsequent in-situ differentiation into either phagocytic monocytes/macrophages in renal tissue, or DC4s in cutaneous lesions. The contribution of these immune cells to tissue damage versus repair, their role as disease initiators versus amplifiers, and consequently their potential as therapeutic targets, remain important areas for future investigation.

## A brief word on the microbiome

Advanced sequencing technologies have also revolutionized the study of the intestinal microbiome both in health and disease states, including SLE [126–129]. While comprehensive reviews of this rapidly evolving field are available elsewhere [127], we highlight here several recent discoveries that implicate the gut microbiome in SLE pathogenesis.

Longitudinal profiling of gut microbiota communities in patients with SLE and HC demonstrated significant temporal instability in their composition among patients, with transient expansions or ‘blooms’ of potentially pathogenic bacterial strains during times of flare. Importantly, the bacterial strain *ruminococcus gnavus* (RG) was found to be highly associated with disease flares in a subset of patients with LN [130]. The highly immunogenic nature of RG’s membrane lipoglycans and the discovery of SLE serum IgG that specifically recognizes these bacterial components suggest a mechanistic role in disease exacerbations [130, 131].

Another emerging area of investigation centers on gut associated lymphoid tissue (GALT) as a microenvironment promoting anti-DNA responses in lupus, potentially triggered by DNA from sampled intestinal bacteria. Immunophenotypic characterization of GALT structures has revealed enrichment of atypical or DN2 B cells, known precursors to antibody secreting cells in SLE. Elevated expression of DNASE1L3 and C1q, proteins with known relevance to SLE pathogenesis, has also been described [132].

These insights provide compelling new paradigms to study potential triggers of autoimmunity in lupus. With ongoing technological advances, the microbiome is an active and fertile area of study that is likely to have tremendous impact in our understanding of disease pathogenesis.

## Conclusions and therapeutic implications

Recent breakthroughs in understanding innate immunity’s role in lupus pathogenesis—through both genetic discoveries and detailed immunophenotyping—illuminate disease heterogeneity and underscore the need for precision medicine approaches. Recent SLE clinical trials have enrolled patients based solely on fulfillment of classification criteria, without stratification by genetic background or immunologic phenotypes beyond limited IFN scores. This approach has resulted in numerous promising therapeutic candidates failing to demonstrate efficacy, while the few approved treatments continue to be prescribed through inefficient trial-and-error approaches.

The identification of specific monogenic SLE-causing variants and risk alleles provides an opportunity to select patients with shared pathophysiologic mechanisms for targeted clinical trials. For instance, patients harboring mutations or risk alleles in TLR7, UNC93B1, or NCF1/2 may demonstrate enhanced responsiveness to therapeutics targeting TLR7 signaling pathways, such as TLR antagonists or IRAK4 inhibitors [133, 134]. In cases where TLR7 GOF primarily drives B-cell intrinsic effects, B-cell depleting therapies—including emerging CAR-T cell approaches—represent rational interventions. Although rituximab failed to meet primary endpoints in phase III SLE trials, the second-generation B-cell depleting agent obinutuzumab recently demonstrated efficacy in LN in phase III studies and may soon receive regulatory approval for this indication, while several CAR-T cell products are advancing through clinical trials [135–137]. Alternatively, patients with documented inflammasome activation may be considered for clinical trials using combination therapies incorporating IL1 blockade, an approach approved for rheumatoid arthritis and autoinflammatory diseases, but not yet formally studied in SLE. Furthermore, better understanding of the role of environmental contributions to SLE pathogenesis, such as the gut microbiome, may lead to systematic longitudinal surveillance and targeted therapeutic manipulation.

In the end, complementary strategies based on genetic stratification, pathway-specific biomarkers, and environmental modification collectively promise to transform the SLE landscape through rational and personalized interventions targeting specific pathogenic mechanisms in individual patients.
